# Nine Metachronous Cancers Primarily Involving the Head and Neck: A Case Report

**DOI:** 10.7759/cureus.104078

**Published:** 2026-02-22

**Authors:** Atsushi Miyauchi, Shuichi Matsumoto, Hiroaki Ito, Masamitsu Hyodo, Masanori Teshima

**Affiliations:** 1 Otolaryngology - Head and Neck Surgery, Kochi University, Nankoku, JPN; 2 Otolaryngology, Hosogi Hospital, Kochi, JPN

**Keywords:** field cancerization, head and neck cancer, metachronous cancers, multicentric zone, multiple primary cancer

## Abstract

A 62-year-old man presented with multiple malignant tumors, predominantly involving the head and neck region, including the esophagus, gum, oropharynx, larynx, lungs, and stomach. Histopathological analysis revealed that while the majority of the tumors were squamous cell carcinomas, other types including spindle cell carcinoma, mucoepidermoid carcinoma, and adenocarcinoma were also identified. Oropharyngeal cancer and oral cancer developed within a previously irradiated field from treatment 10 years prior. All subsequent primary tumors were detected at early stages through extensive multimodal surveillance, allowing for minimally invasive curative resections and contributing to long-term survival. This case underscores the necessity of lifelong, continuous monitoring for survivors of multiple primary cancers, as new malignancies can emerge long after initial treatments.

## Introduction

Advances in cancer treatment and early detection have increased the risk that cancer survivors will develop a second primary cancer [1]. This case report aims to examine the prevalence of second primary cancers in patients with head and neck cancers. Multiple primary cancers are defined according to modified Warren-Gates criteria, where each tumor should present a definite picture of malignancy, each tumor should be histologically distinct with at least 2 cm normal mucosa in between or duration of five years, the possibility that one is a metastasis of other must be excluded, and, additionally, tumors in an interval of more than six months are defined as metachronous [2]. Here, we present the case of a patient who developed nine metachronous primary cancers over 18 years. To our knowledge, such a high number of tumors is scarce; they primarily affected the head, neck, and esophageal regions. This case highlights the clinical importance of lifelong, multi-organ surveillance in patients with field cancerization, demonstrating that even with a high frequency of primary cancers, early detection and appropriate intervention can lead to long-term survival.

## Case presentation

**Figure 1:**
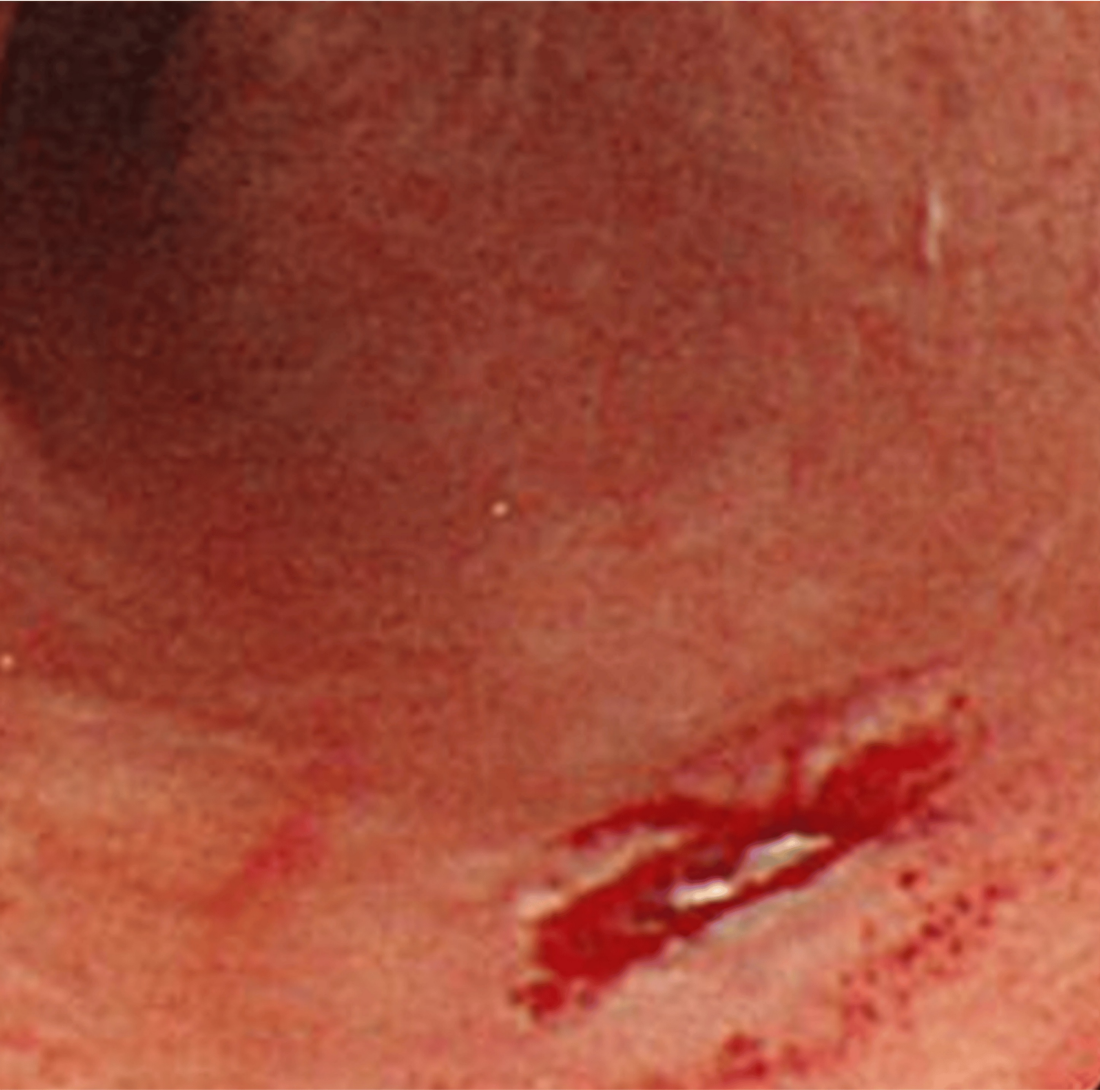
First esophageal cancer Located 26 cm from the incisor

The first cancer was found in 2000, when a 62-year-old man had a health check with an upper gastrointestinal endoscopy, which revealed an esophageal lesion that was diagnosed as esophageal cancer, tumor in situ (Tis) (Figure 1).

The patient had a history of silicosis (due to tunnel work), vibration disease, and 98 pack-years of smoking and 108 g/day of alcohol. Endoscopic mucosal resection was performed. Thereafter, the patient was followed up with upper gastrointestinal endoscopy every few months. Although the esophageal cancer recurred three times over a four-year period, all lesions were superficial and successfully treated with curative endoscopic resection.

**Figure 2:**
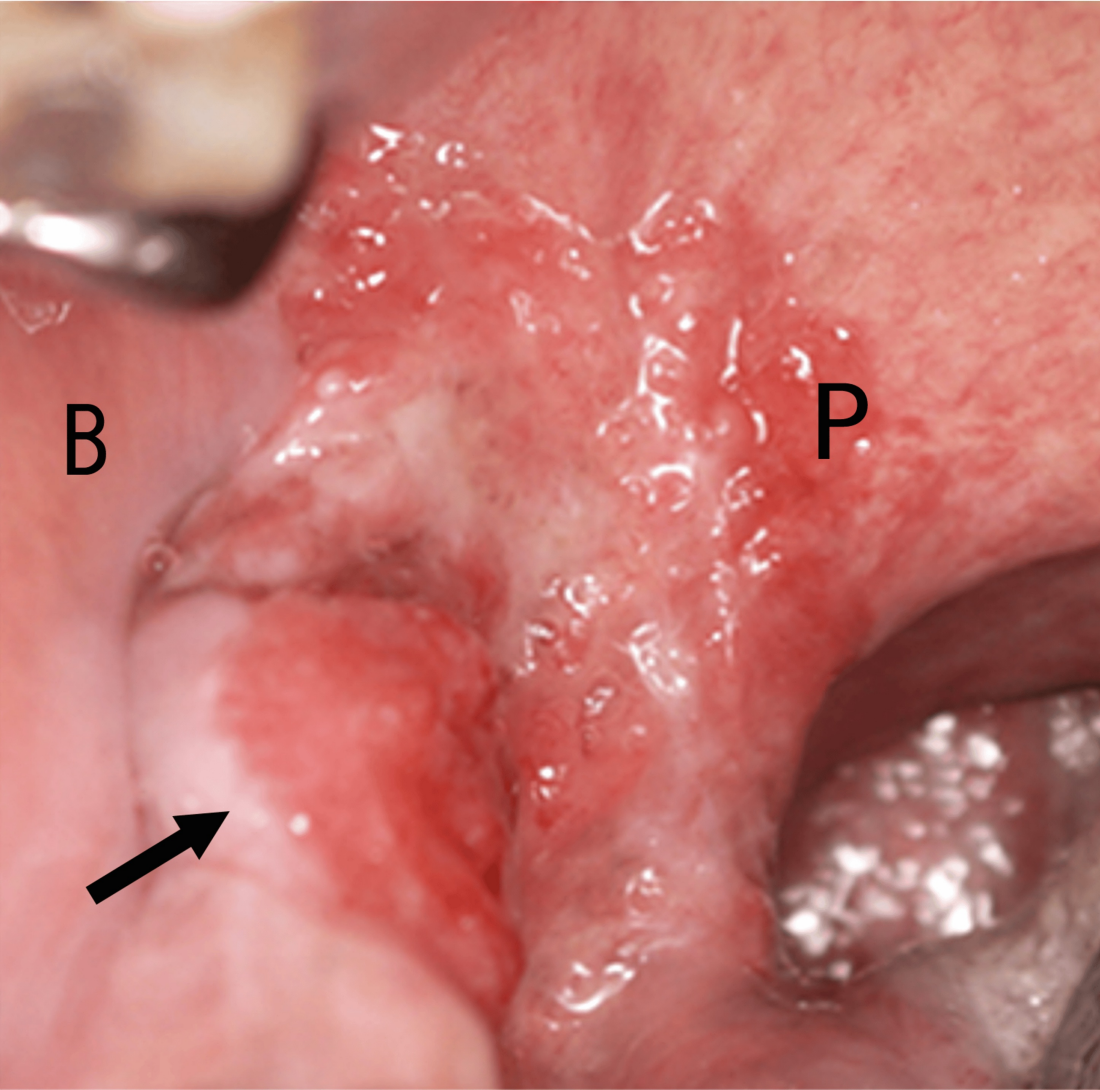
Second oral cancer: T2 Right lower gingiva (indicated by arrow), B: right buccal  P: right palatoglossal arch

The second cancer was found in 2001, when the patient had a leukoplakia on the right lower gingiva, which had been bothering him for a year. As the lesion gradually became erythematous and associated with intermittent localized pain, the patient visited the oral and maxillofacial surgery department in our hospital. Biopsy confirmed oral squamous cell carcinoma as the second oral cancer, T2N0M0, stage II (Figure 2).

The patient underwent chemoradiotherapy. Radiation fields were applied from the lower of maxillary sinus to hyoid bone and performed with 6 MV X-rays total 40 Gy (2 Gy/fraction, once a day, five times a week for four weeks). As concomitant chemotherapy, two cycles of carboplatin (300mg for the first cycle and 600mg for the second cycle), oral S-1 (120mg/day) for four weeks, and bleomycin 5 mg two times a week (eight cycles; total dose 40 mg). The disease was completely cured.

**Figure 3:**
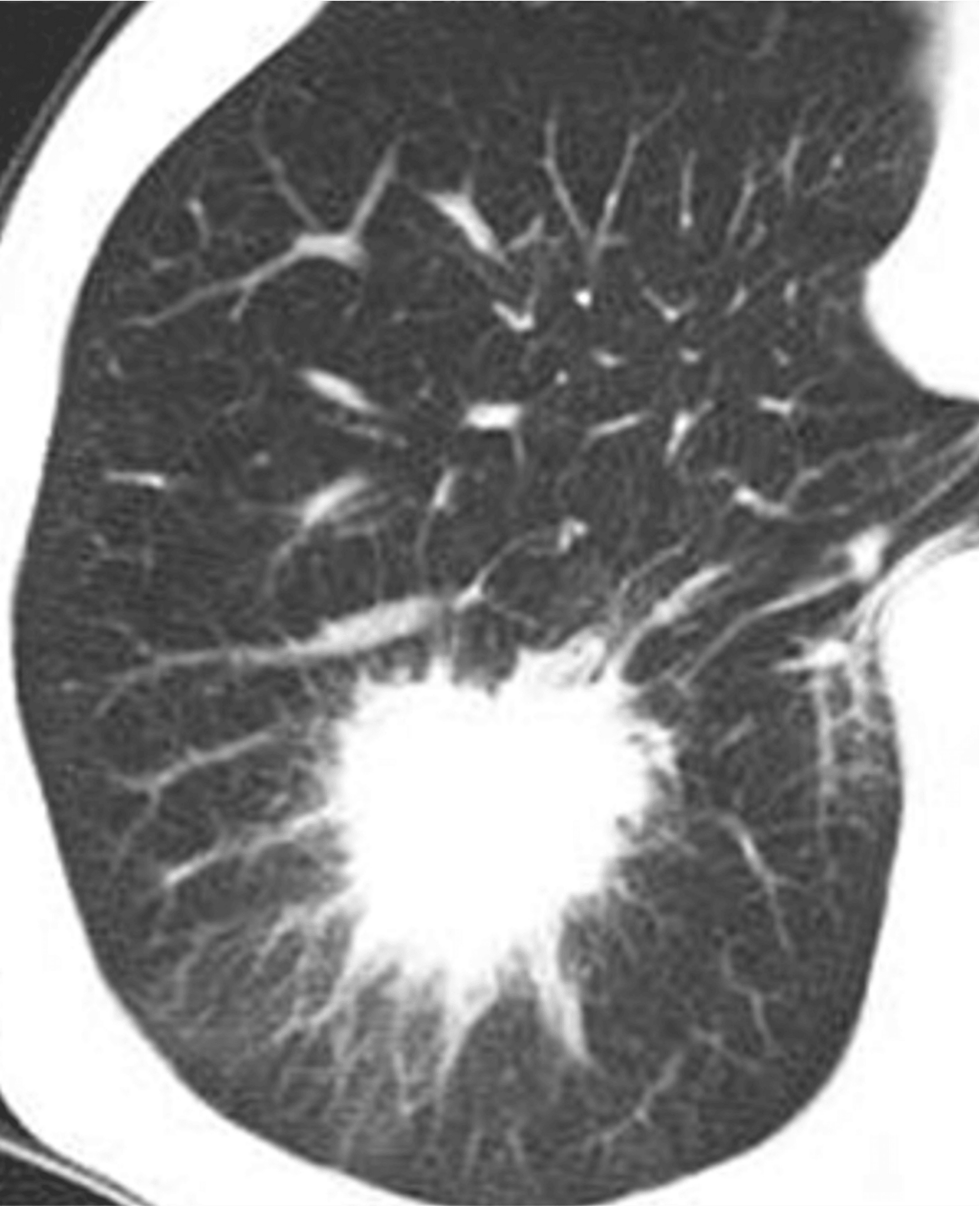
Third lung cancer: T2 Right lower lobe, characterized by a 45 mm mass lesion

The third cancer was found in 2006. The patient had a CT scan during a routine check-up, which revealed an abnormal shadow in the right lower lung. The patient had no respiratory symptoms, and biopsy under bronchoscopy confirmed squamous cell carcinoma (Figure 3).

This lesion was considered a newly metachronous third lung cancer, T2N0M0, stage I-B, because five years had passed since the second oral cancer was cured, and the lesion was sporadic and spiculated CT finding. The right lower lobe was resected, and adjuvant chemotherapy with tegafur-uracil (400 mg/day) was administered orally for two years. Thereafter, the patient was followed up with regular clinical examinations, and CT scan and PET-CT were added to monitor for any signs of frequent recurrence or the development of multiple primary cancers.

**Figure 4:**
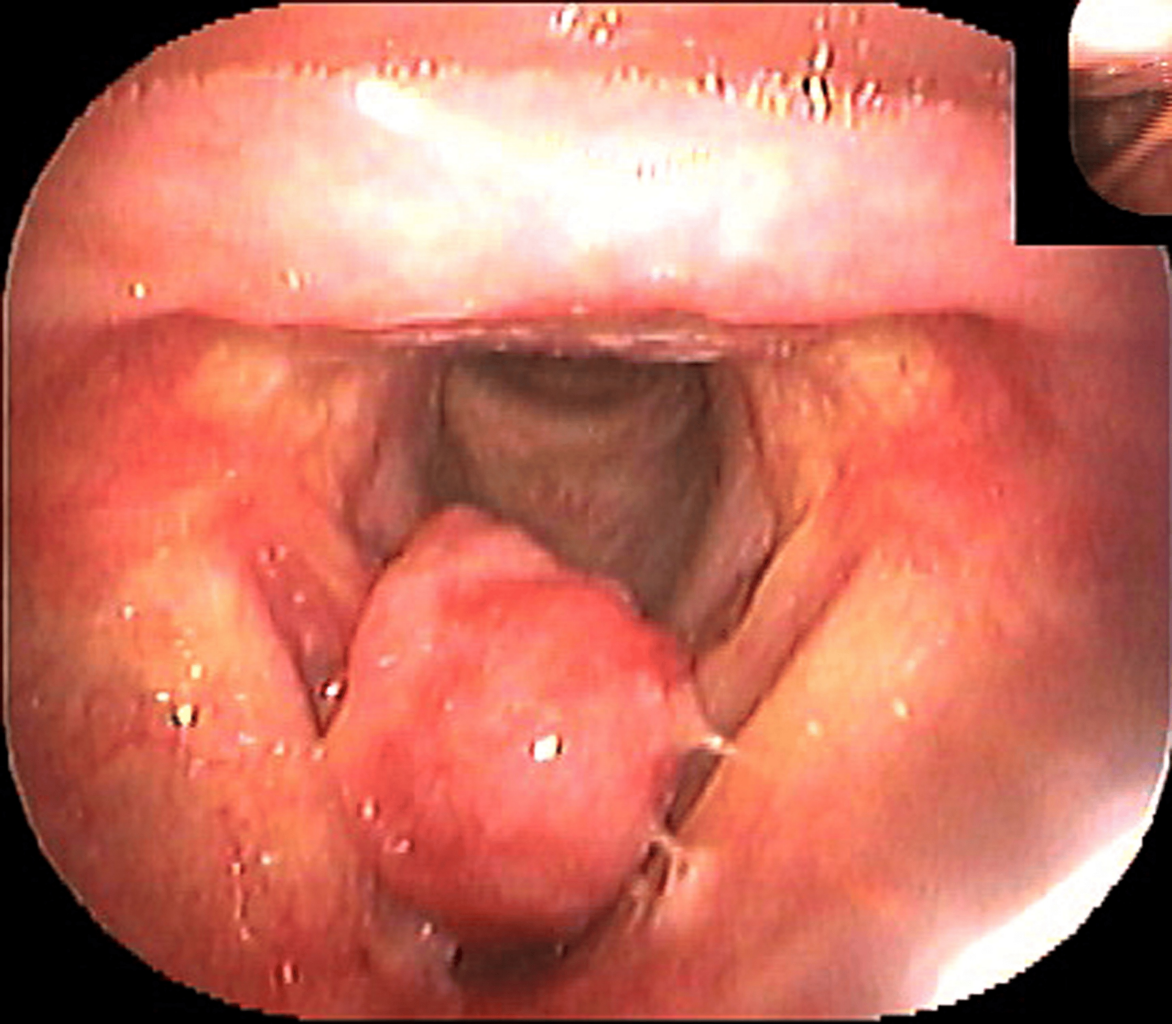
Fourth laryngeal cancer: T1 Right vocal cord

The fourth cancer was found in 2010, when the patient had a slight hoarseness and sore throat for several months. A few months prior to visiting our department, the patient had a loss of appetite and worsening hoarseness. An upper gastrointestinal endoscopy examination performed at a local clinic revealed a vocal tumor. For further evaluation and treatment, the patient was referred to our department. A pedunculated mass was identified on the right vocal cord (Figure 4).

The tumor was resected under direct rigid laryngoscopy, and histopathological diagnosis confirmed spindle cell carcinoma, as the fourth cancer, T1aN0M0, stage I. Recurrence was observed five months later, necessitating vertical laryngectomy, and disease was completely cured.

**Figure 5:**
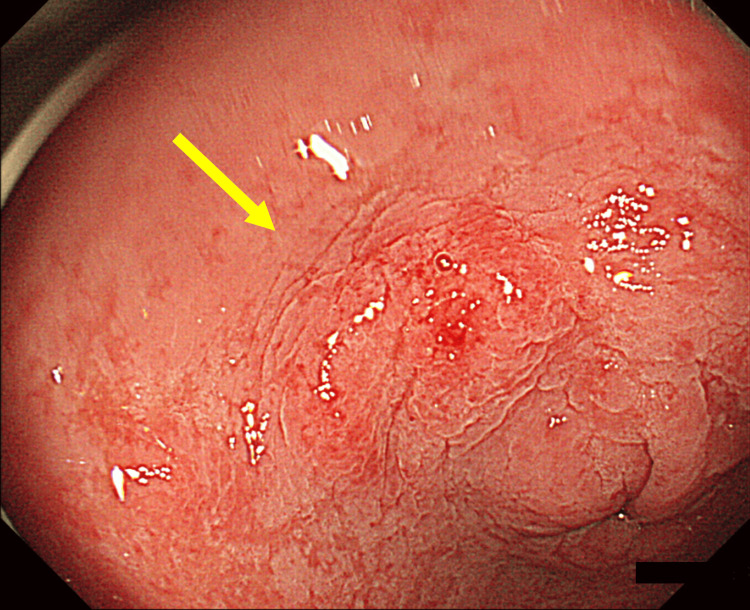
Fifth stomach cancer: T1a Tumor in the antrum (indicated by arrow)

The fifth cancer was nine months later in 2010, when, after the initial diagnosis of the fourth cancer, an upper gastrointestinal endoscopy during a routine check-up for esophageal cancer identified early gastric cancer (Figure 5).

The disease was resected endoscopically, and histopathology diagnosed it as adenocarcinoma, T1aN0M0, stage I.

**Figure 6:**
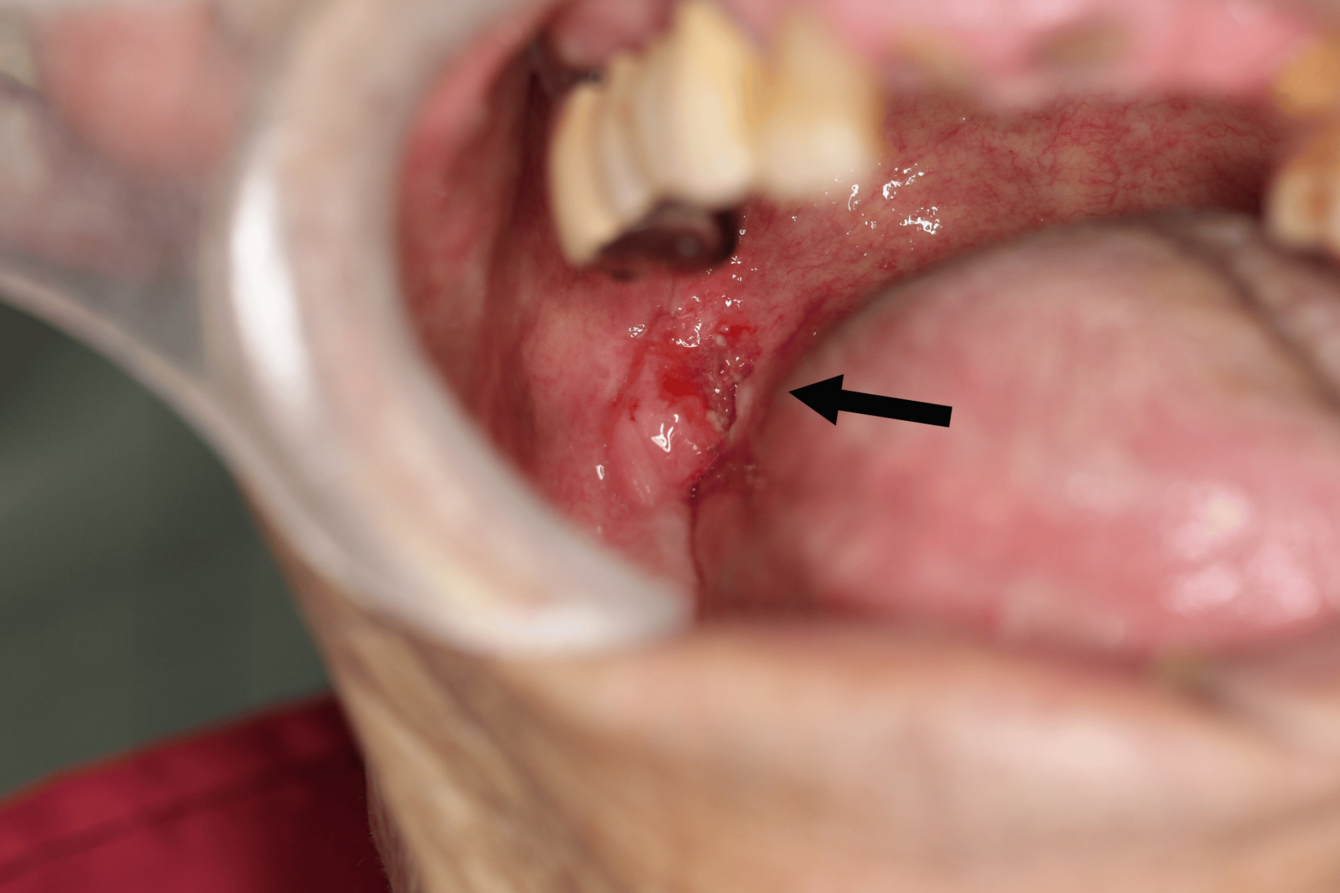
Sixth oropharyngeal cancer: T1 Right soft palate (indicated by arrow)

In 2012, a routine upper gastrointestinal endoscopy revealed an ulcerative lesion in the right lower aspect of the soft palate. The patient had no symptoms and no local pain. A biopsy was performed, and it was diagnosed as oropharyngeal squamous cell carcinoma, as the sixth cancer, T1N0M0, stage I (Figure 6). The lesion was resected via a transoral approach with CO2 laser.

**Figure 7:**
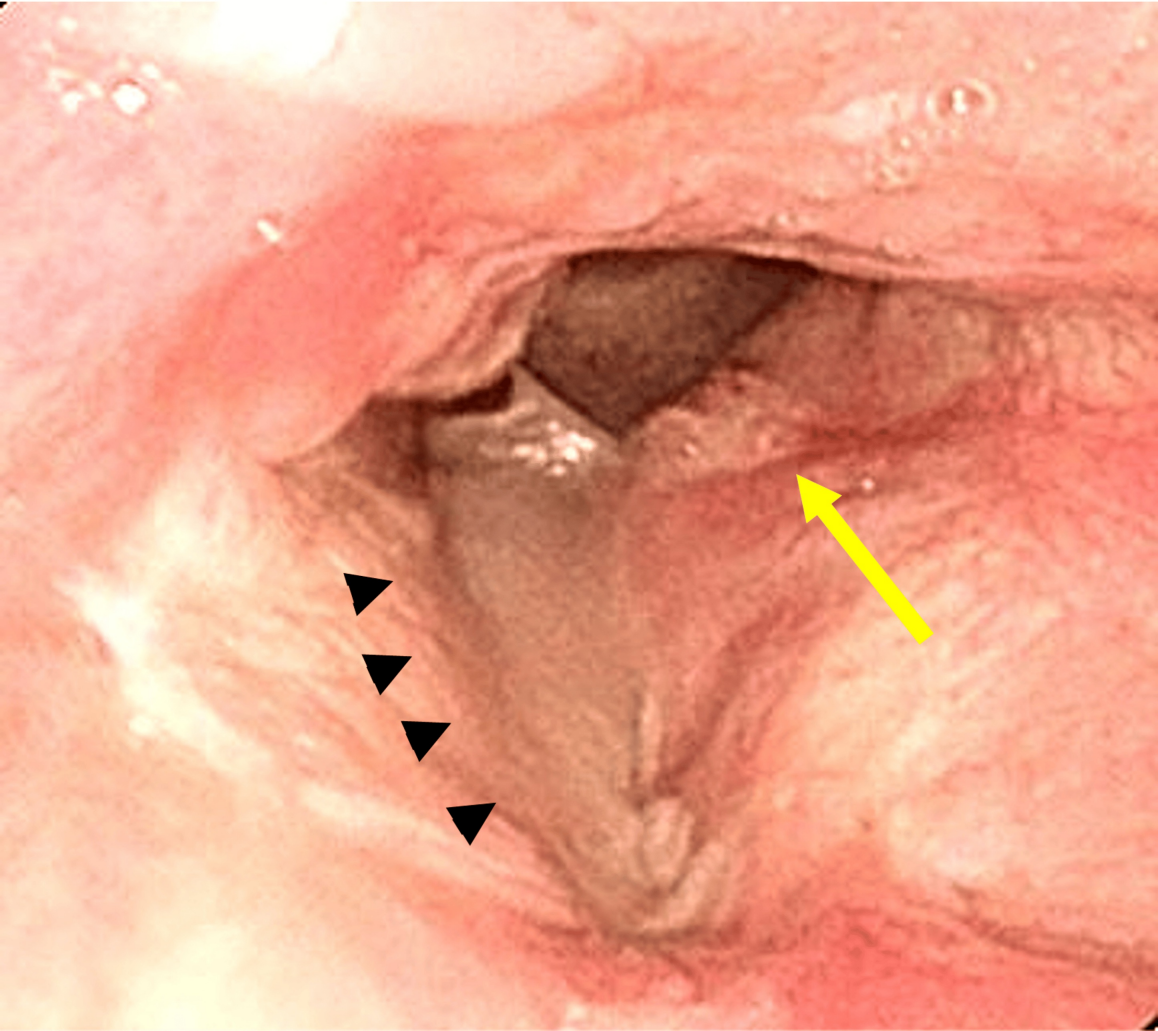
Seventh laryngeal cancer: T1a Tumor of left vocal cord (indicated by arrow), Right vocal cord has been replaced skin graft (arrow head) by vertical laryngectomy for recurrent of fourth laryngeal cancer

The seventh cancer was found in 2013, when the patient had a PET-CT scan during a routine check-up that revealed an abnormal shadow at the left larynx, and a tumor was detected in the left vocal cord with a laryngoscope (Figure 7).

The patient had no symptoms with hoarseness and local pain. The lesion was suspicious for recurrence of the fourth laryngeal cancer and was surgically resected. Histopathology confirmed it as squamous cell carcinoma. This lesion was identified as a newly metachronous seventh laryngeal cancer, T1aN0M0, stage I, because it was histologically distinct from the previous fourth laryngeal cancer (spindle cell carcinoma), and the lesion appeared on the opposite side.

**Figure 8:**
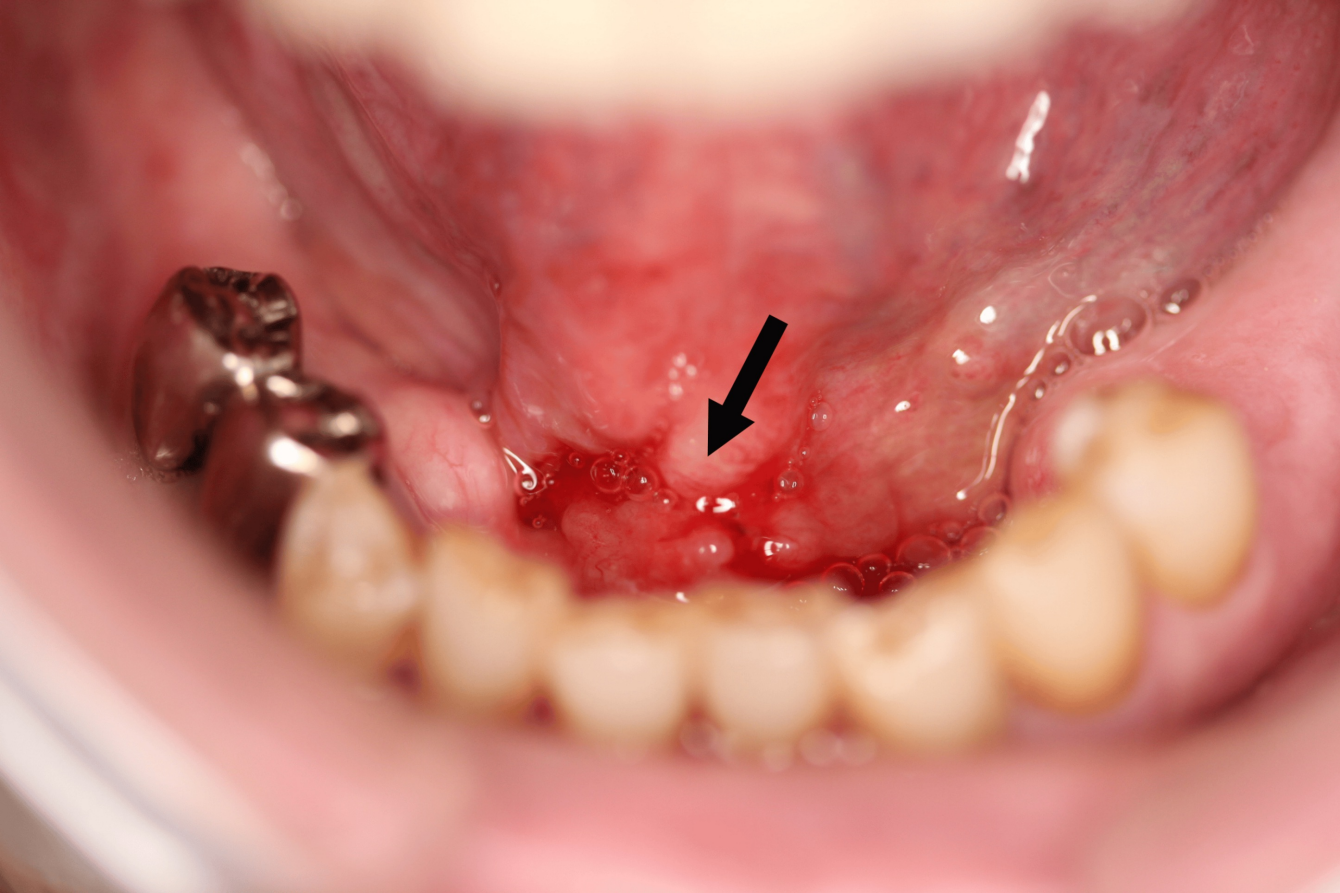
Eighth oral cancer: T1 Floor of the mouth (indicated by arrow)

The eighth cancer was found in 2015. The patient had a PET-CT scan during a routine check-up, which revealed an abnormal shadow at the tip of the tongue, and an ulcerative lesion with induration was found at the median of the oral floor (Figure 8).

The patient had no symptoms and no local pain. A biopsy diagnosed it as mucoepidermoid carcinoma, constituting the eighth metachronous oral cancer, T1N0M0, stage I. The tumor was locally resected with CO2 laser, and histopathological diagnosis was grade III, high grade. One year after the initial surgery, the first local recurrence was identified. The patient underwent marginal mandibulectomy combined with right neck dissection. Six months later (1.5 years after the initial surgery), a second local recurrence occurred. A repeat marginal mandibulectomy was performed to achieve local control. They were all classified as eighth cancer.

**Figure 9:**
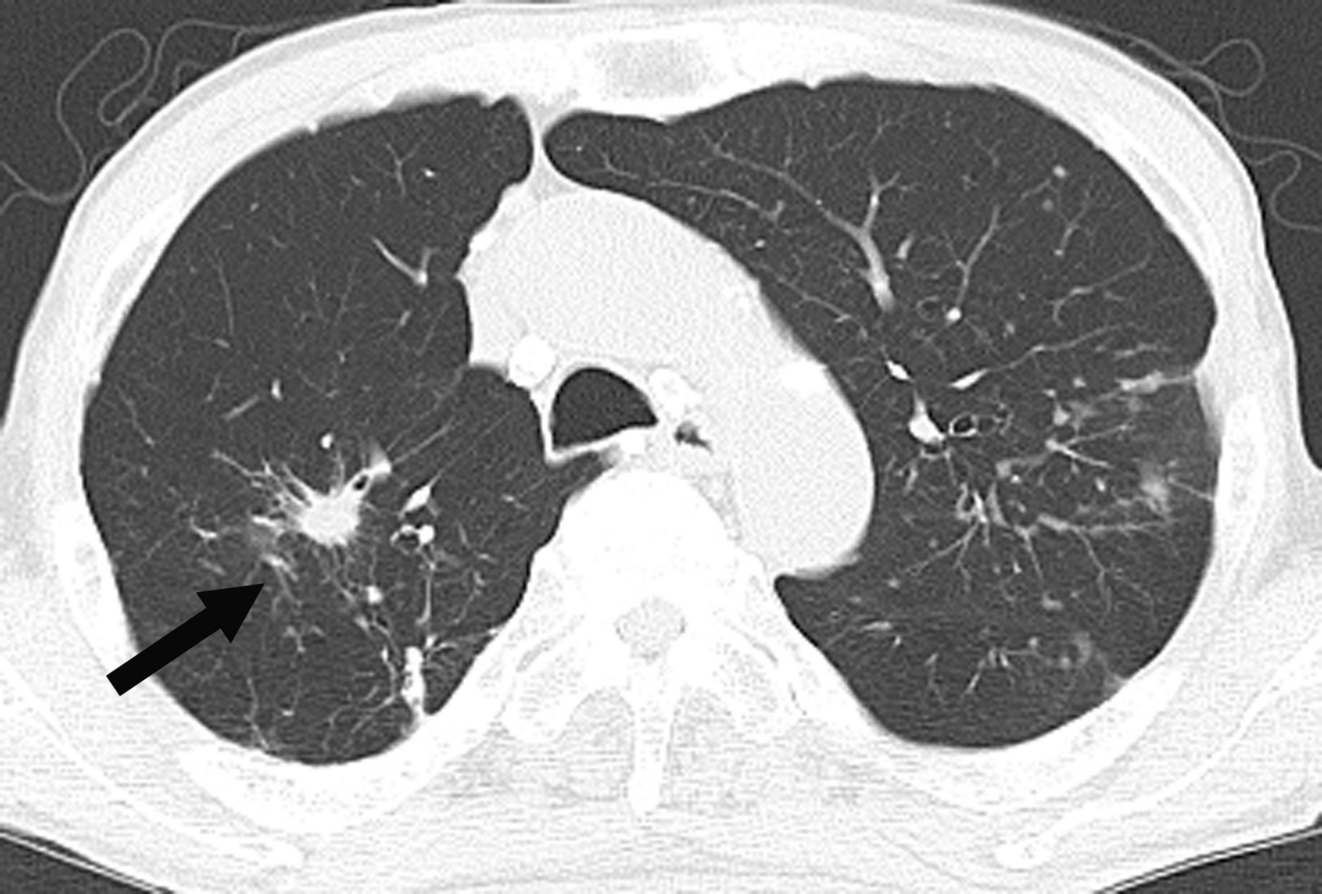
Ninth lung cancer: T1a Right upper lobe, 12 mm spiculated mass lesion (indicated by arrow)

The ninth cancer was found in 2017, when a routine CT checkup for screening of recurrence and new metastasis for prior metachronous cancers revealed a lung tumor in the right upper lobe (Figure 9).

**Figure 10:**
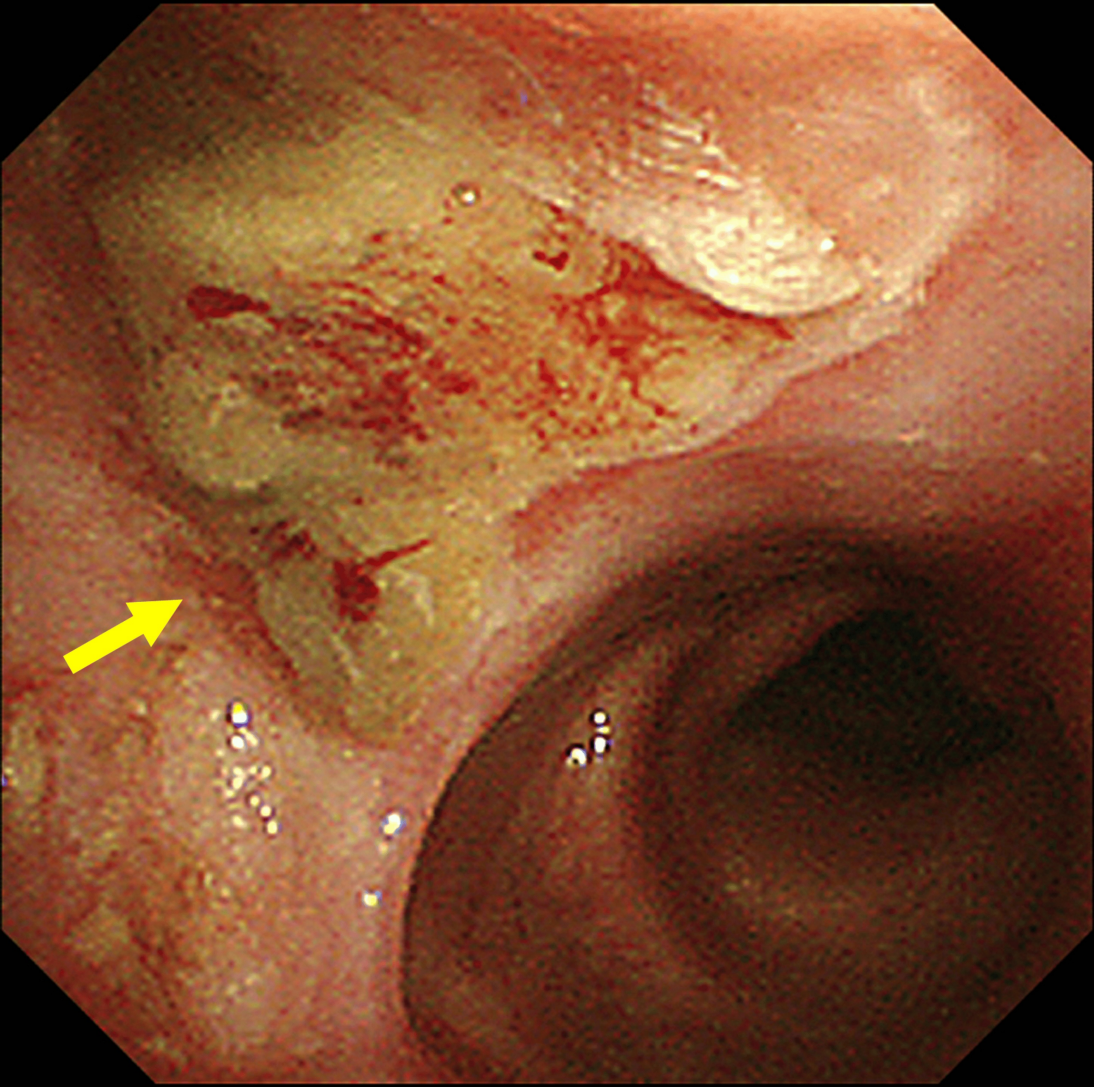
Bronchoscopic findings showing the ninth lung tumor Tumor (indicated by arrow) was originated from bronchial epithelium.

The patient had no respiratory symptoms with hemoptysis. Bronchoscope biopsy confirmed squamous cell carcinoma. This lesion was considered a newly metachronous ninth lung cancer, T1aN0M0, stage I, because more than 10 years had passed since the third right lung cancer, the tumor was located inside the bronchus and originated from bronchial epithelium under bronchoscopy (Figure 10) and the CT finding was sporadic and spiculated pattern. The right upper lobectomy was performed. Seven months later, local recurrence occurred. As systemic chemotherapy, two cycles of carboplatin (AUC 6) and nab-paclitaxel (100 mg/m²) were administered, followed by six cycles of pembrolizumab (200 mg). One year and six months after the recurrence, the patient succumbed to the ninth lung cancer, approximately 18 years after the first cancer (Table 1).

**Table 1:** Clinical Course

	Interval from 1st cancer	Site	Location	Diagnosis	TNM	Pathology	Treatment	Outcome
1st	62-year-old	Esophagus	Thoracal	Gastrointestinal endoscope	Tis	Squamous cell carcinoma	Endoscopic resection	Recurrence three times within four years: Endoscopic resection of each
2nd	1Y	Oral	Right lower gingiva	Clinical finding	T2N0M0	Squamous cell carcinoma	Chemoradiotherapy: Radiation 40Gy Chemotherapy: CBDCA 900mg, S-1 120mg 4 weeks, peplomycin 40mg	
3rd	6Y	Lung	Right lower lobe	CT scan	T2N0M0	Squamous cell carcinoma	Surgery: Right lower lobectomy adjuvant chemotherapy: UFT 400mg/day for two years	
4th	10Y	Larynx	Right vocal cord	Laryngoscope	T1aN0M0	Spindle cell carcinoma	Surgery: Resection direct laryngoscopy	Recurrence: Vertical partial laryngectomy
5th	10Y9M	Stomach	Antrum	Gastrointestinal endoscope	T1aN0M0	Adenocarcinoma	Endoscopic resection	
6th	12Y6M	Oropharynx	Right soft palate	Gastrointestinal endoscope	T1N0M0	Squamous cell carcinoma	Surgery: Transoral CO2 laser resection	
7th	13Y6M	Larynx	Left vocal cord	PET-CT	T1aN0M0	Squamous cell carcinoma	Surgery: CO2 laser resection direct laryngoscopy	
8th	15Y7M	Oral	Floor of mouth	PET-CT	T1N0M0	Mucoepidermoid carcinoma, Grade III (High grade)	Surgery: Transoral CO2 laser resection	Recurrence twice: 1Y: First recurrence: Marginal mandibulectomy, right neck dissection; 1Y6M: Second recurrence: Marginal mandibulectomy
9th	17Y1M	Lung	Right upper lobe	CT scan	T1aN0M0	Squamous cell carcinoma, Grade 3	Surgery: Right upper lobectomy	Recurrence: Chemotherapy CBDCA (6AUC) and nab-paclitaxel (100mg/m^2^): two cycles followed by pembrolizumab 200mg: six cycles
	18Y7M							Died of ninth lung cancer recurrence, 18 years after the first cancer.

## Discussion

The incidence of a second primary cancer following an initial diagnosis of cancer is approximately 5 to 10%, which is 1.1 to 1.4-fold higher than in the general population [3-5]. This ratio varies depending on the location of the initial cancer: it is lower than 10% if the initial lesions are liver, pancreatic, and breast cancers, but higher than 10% if colorectal and prostate cancers. Moreover, if oral or pharyngeal lesion, the risk increases by more than 20-fold [3]. Maier et al. reported that when the tobacco index was greater than 50 pack-years, the risk of head and neck cancer increased to 12-to-70-fold, and greater than 75 g daily alcohol intake increased to 12-to-16-fold [6]. This elevated risk in the head and neck regions is explained by the concept of field cancerization [7]: widespread exposure to a carcinogen leads the epithelium to cancer preconditioned change across these areas. Over time, irreversible change progresses to malignancy at multiple points, resulting in metachronous cancers. Furthermore, the molecular analyses have detected genetic alterations in more than 30% of samples in clinically normal mucosa adjacent to head and neck primaries [8]. Vrabec proposed the concept of a 'multicentric zone' encompassing the oral cavity, pharynx, larynx, esophagus, stomach, and lung [9]. Atkinson et al. cited chronic exposure to irritants such as smoking and alcohol as common carcinogenic factors in these ‘multicentric zone’, contributing to the recurrence of multiple secondary cancers [10]. Sung et al. and Chen et al. also found the primary and secondary cancers to occur together within the multicentric zone, with an increased risk ranging from several-fold up to 20-fold for both the initial and subsequent cancers, such as lung, laryngeal and esophageal malignancies [4,11]. In this patient, the lifestyle of 98 pack-years of tobacco and around 108 g daily alcohol strongly implicates these exposures, and multiple metachronous cancers occurred in as many as eight locations within a multicentric zone.

Approximately 40-70% of secondary cancers are diagnosed within five years from the primary cancer diagnosis [3-5], and especially, the first year has the highest incidence of secondary cancers, followed by a gradual decline over time [9]. However, new primaries can still occur more than a decade later, emphasizing the need for vigilance during both the early post-treatment period and the long term. In this patient, while the interval from the second oral cancer to the third lung cancer exceeded five years, others were diagnosed within five years, besides four cases within one year. Owing to careful follow-up, each secondary cancer was detected in early T stage and treated with minimally invasive surgery, supporting prospects for extended survival. Paradoxically, the success of early diagnosis and treatment will eventually lead to an increasing incidence of newly identified multiple primary malignancies; consequently, the occurrence of multiple primary cancers, as seen in this case, will become more common.

Genetic susceptibility may also play a role. Among aldehyde dehydrogenase-2 (ALDH2) polymorphisms, the heterozygous and homozygous deficient variants are associated with facial flushing after consuming small amounts of alcohol. This phenotype is linked to field cancerization in the upper aerodigestive tract [12]. This patient reported flushing, suggesting ALDH2 deficiency. Microsatellite instability and reduced p53 expression have also been linked to multiple primaries [13]. Li-Fraumeni syndrome, driven by germline TP53 alterations, has been reported in two cases involving nine primary tumors, including osteosarcoma, lung cancer, gastric cancer, and leukemia over 20-40 years [14,15]. Several other cases with TP53 germ line mutations have been documented [16], involving sarcoma, brain, leukemias, and other organs. Although genetic testing was not performed in this case and field cancerization due to lifestyle factors within the multicentric zone was prominent, genetic evaluation should be considered in cases of such a high number of primary malignancies.

Radiation-induced cancers are difficult to diagnose clinically. Generally, radiation-induced leukemia occurs five to nine years after exposure and solid tumors often develop 10 years or more [1]. Therefore, a solid tumor should be considered as radiation-induced if it appears more than 10 years after in a previously irradiated area. In the present case, both the sixth oropharyngeal cancer and the eighth oral cancer met the clinical criteria for radiation-induced malignancies associated with the prior chemoradiotherapy for second oral cancer. However, the pathogenesis of the oropharyngeal cancer may also be attributed to the patient’s long-standing lifestyle habits (tobacco and alcohol consumption). Regarding the oral cancer, although it is known that such tumors can arise from minor salivary glands in the oral cavity, it was histologically identified as mucoepidermoid carcinoma - a subtype generally recognized as having a potential association with prior radiation exposure [17]. Even when a patient has a history of radiation therapy, careful attention should be paid to the development of secondary cancers within irradiated fields, even after a latency period exceeding 10 years.

## Conclusions

This patient presented with multiple malignant tumors, mainly in the head and neck area, including cancers of the esophagus, gum, oropharynx, larynx, lungs, and stomach. The histology included squamous cell carcinoma, spindle cell carcinoma, mucoepidermoid carcinoma, and adenocarcinoma. According to the locations, histological types, and timing of diagnosis, mostly the disease suggests influences from field cancerization for smoking, alcohol, and radiation-related carcinogenesis. All secondary primary tumors were detected early stage and treated with curative resection. Extensive multimodal surveillance enabled early detection and intervention, supporting long-term survival, and strict follow-up proved advantageous especially in head and neck fields. During routine post-treatment monitoring after an initial cancer diagnosis, clinicians should recognize characteristic patterns and high-risk locations for secondary primary cancers, and prolonged, lifelong monitoring is essential.
